# Biochemical Markers of Musculoskeletal Health and Aging to be Assessed in Clinical Trials of Drugs Aiming at the Treatment of Sarcopenia: Consensus Paper from an Expert Group Meeting Organized by the European Society for Clinical and Economic Aspects of Osteoporosis, Osteoarthritis and Musculoskeletal Diseases (ESCEO) and the Centre Académique de Recherche et d'Expérimentation en Santé (CARES SPRL), Under the Auspices of the World Health Organization Collaborating Center for the Epidemiology of Musculoskeletal Conditions and Aging

**DOI:** 10.1007/s00223-022-01054-z

**Published:** 2023-01-12

**Authors:** Aurélie Ladang, Charlotte Beaudart, Jean-Yves Reginster, Nasser Al-Daghri, Olivier Bruyère, Nansa Burlet, Matteo Cesari, Antonio Cherubini, Mario Coelho da Silva, Cyrus Cooper, Alfonso J. Cruz-Jentoft, Francesco Landi, Andrea Laslop, Stefania Maggi, Ali Mobasheri, Sif Ormarsdottir, Régis Radermecker, Marjolein Visser, Maria Concepcion Prieto Yerro, René Rizzoli, Etienne Cavalier

**Affiliations:** 1 Department of Clinical Chemistry, CHU de Liège, University of Liège, Liège, Belgium; 2 Division of Public Health, Epidemiology and Health Economics, WHO Collaborating Center for Public Health Aspects of Musculo-Skeletal Health and Ageing,, University of Liège, Liège, Belgium; 3 Biochemistry Department, College of Science, Chair for Biomarkers of Chronic Diseases, King Saud University, Riyadh, 11451 Saudi Arabia; 4 Department of Clinical Sciences and Community Health, University of Milan, Milan, Italy; 5 Geriatric Unit, Fondazione IRCCS Ca’ Granda Ospedale Maggiore Policlinico, Milan, Italy; 6 Geriatric Unit, IRCCS Istituti Clinici Scientifici Maugeri, Milan, Italy; 7 Laboratory of Clinical and Therapeutical Pharmacology, Lisbon, Portugal; 8 MRC Lifecourse Epidemiology Unit, University of Southampton, Southampton, UK; 9 Servicio de Geriatría. Hospital Universitario Ramón Y Cajal (IRYCIS), Madrid, Spain; 10 Department of Geriatrics, Neurosciences and Orthopedics, Catholic University of the Sacred Heart, Rome, Italy; 11 Scientific Office, Federal Office for Safety in Health Care, Vienna, Austria; 12 CNR Aging Branch-IN, Padua, Italy; 13 State Research Institute Centre for Innovative Medicine, Vilnius, Lithuania; 14 Research Unit of Medical Imaging, Physics and Technology, Faculty of Medicine, University of Oulu, Oulu, Finland; 15 Department of Joint Surgery, The First Affiliated Hospital of Sun Yat-Sen University, Guangzhou, China; 16 Landspitali, University Hospital of Iceland, Reykjavik, Iceland; 17 Department of Diabetes, Nutrition and Metabolic Disorders, Clinical Pharmacology, University of Liege, CHU de Liège, Liège, Belgium; 18 Department of Health Sciences, Vrije Universiteit Amsterdam, Amsterdam, the Netherlands; 19 Agencia Española de Medicamentos Y Productos Sanitarios, Madrid, Spain; 20 Faculty of Medicine, Service of Bone Diseases, Geneva University Hospitals, Geneva, Switzerland

**Keywords:** Sarcopenia, Biomarkers, Biochemical markers, Recommendations, Clinical trial, Pharmacological drugs

## Abstract

In clinical trials, biochemical markers provide useful information on the drug’s mode of action, therapeutic response and side effect monitoring and can act as surrogate endpoints. In pharmacological intervention development for sarcopenia management, there is an urgent need to identify biomarkers to measure in clinical trials and that could be used in the future in clinical practice. The objective of the current consensus paper is to provide a clear list of biochemical markers of musculoskeletal health and aging that can be recommended to be measured in Phase II and Phase III clinical trials evaluating new chemical entities for sarcopenia treatment. A working group of the European Society for Clinical and Economic Aspects of Osteoporosis, Osteoarthritis and Musculoskeletal Diseases (ESCEO) proposed classifying biochemical markers into 2 series: biochemical markers evaluating musculoskeletal status and biochemical markers evaluating causal factors. For series 1, the group agreed on 4 biochemical markers that should be assessed in Phase II or Phase III trials (i.e., Myostatin-Follistatin, Brain Derived Neurotrophic Factor, *N*-terminal Type III Procollagen and Serum Creatinine to Serum Cystatin C Ratio – or the Sarcopenia Index). For series 2, the group agreed on 6 biochemical markers that should be assessed in Phase II trials (i.e., the hormones insulin-like growth factor-1 (IGF-I), dehydroepiandrosterone sulphate, and cortisol, and the inflammatory markers C-reactive protein (CRP), interleukin-6 and tumor necrosis factor-*α*), and 2 in Phase III trials (i.e., IGF-I and CRP). The group also proposed optional biochemical markers that may provide insights into the mode of action of pharmacological therapies. Further research and development of new methods for biochemical marker assays may lead to the evolution of these recommendations.

## Background

Sarcopenia is a progressive and generalized skeletal muscle disorder defined by low levels of measures of muscle strength, muscle quantity/quality and physical performance [1]. Sarcopenia is related to important negative clinical outcomes, with a series of delicate economic and social implications, including impaired mobility, loss of quality of life, institutionalization, hospitalization and death [2–6]. Given these critical consequences linked to sarcopenia, several pharmacological interventions have been studied in the past few years to understand whether they might be effective for sarcopenia. In April 2020, no fewer than 44 interventional trials studying pharmacological interventions for sarcopenia were indexed in the clinicaltrials.gov database [7]. However, to date, there are still no therapeutic indications for sarcopenia that are accepted by regulatory agencies in the US and Europe [8].

Nevertheless, in consideration of the continued rapid development of therapeutic strategies to slow down the development or reverse the sarcopenia process, it has become urgent to address issues related to the optimal conduct of clinical trials in this area, in particular, the identification of biomarkers to be measured in trials. A biomarker is defined as “*a characteristic that is objectively measured and evaluated as an indicator of normal biologic processes, pathogenic processes, or pharmacologic responses to a therapeutic intervention”* [9]. Biomarkers can include soluble analytes measured in biospecimens such as blood or urine and anatomic biomarkers such as muscle mass or muscle strength measurements. This specific report will focus on soluble biomarkers, also called biochemical markers. In drug development and clinical trials, biochemical markers are useful, among other things, to increase knowledge about the mode of action of a drug, monitor the (early) therapeutic response of treatment, monitor side effects and act as potential surrogate endpoints [10]. Biochemical markers are also intended to be correlated with the evolution of “hard” clinical endpoints following treatment, such as loss of mobility and falls. Because of the complexity of sarcopenia, the European Working Group on Sarcopenia in Older People (EWGSOP2), which provides the very latest consensus definition of sarcopenia, suggests the need to develop a panel of biomarkers, as it is unlikely that one single biomarker could be specific enough [1].

To address this topic, the European Society for Clinical and Economic Aspects of Osteoporosis and Osteoarthritis (ESCEO) organized a working group meeting including well-recognized key leaders. The working group members’ expertise and knowledge were shared to gain an understanding of the currently available biochemical markers that could be used in clinical trials of drugs for sarcopenia. The objective of the current consensus paper is to provide a clear list of biochemical markers of musculoskeletal status and the pathophysiological mechanisms of sarcopenia that are recommended in 2022 to be assessed in phase II and phase III clinical trials of drugs aimed at managing sarcopenia.

## Methods

### Literature Reviews and Meeting Consensus

The European Society for Clinical and Economic Aspects of Osteoporosis, Osteoarthritis and Musculoskeletal Disorders (ESCEO ASBL) jointly with the Centre Académique de Recherche et d’Expérimentation en Santé (CARES SRL) organized in September 2022, under the auspices of the World Health Organization Collaborating Center for Epidemiology of Musculoskeletal Health and Aging, a working group including scientists, specialists in laboratory medicine and clinician experts in the field of biochemical markers and sarcopenia as well as representatives of the regulatory bodies. The methodology employed in several other publications emerging from different ESCEO working groups was replicated [7, 11–14]. Three members of the present working group (EC, AL and AM) first prepared, before the meeting, a literature review to identify potential biochemical markers of sarcopenia to be assessed in clinical trials of drugs and possibly in clinical practice in the future. Literature reviews and original studies (either observational or interventional) published until September 2022 were searched on Medline and Scopus using a combination of the following MeSH terms and keywords: (Biomarkers (MeSH) OR [biologic* OR biochemical or laboratory or clinical or serum or surrogate) AND marker*] OR biomarker*) AND (Sarcopenia (MeSH) OR sarcopeni* OR EWGSOP). Additional studies were identified by a manual search of the bibliographies of relevant papers. Experts in the field were also requested to provide additional references. Finally, a free web search on Google Scholar was also performed.

The three members involved in this literature search were asked to prepare a presentation summarizing their findings and to make some preliminary recommendations to be discussed during the meeting.

Then, all experts met during a face-to-face meeting to discuss recommendations for the selection of biochemical markers of musculoskeletal status and the pathophysiological mechanisms of sarcopenia to be assessed in clinical trials of drugs aimed at the management of sarcopenia. The discussion ended when all experts reached a consensus and agreed on the conclusion of the manuscript. The general plan of the manuscript was also discussed and agreed upon by all. The core writing group (AL, CB, RR and EC) provided the first version of the manuscript, and all experts were invited to offer comments and corrections and ultimately approve its contents.

### Clinical and Analytical Performances of Biochemical Markers

To be recommended in this paper, the biochemical markers examined needed to encounter clinical evidence as well as analytical performance. Among clinical evidence, the considered biochemical markers were required to be either increased or decreased in patients suffering from sarcopenia or either modified by non-pharmacological strategies aimed at sarcopenia management in older adults. Biochemical markers showing correlations with outcome that are available for phase II studies aimed at sarcopenia management as defined in our previous ESCEO recommendations [7] were also considered. Additionally, the selected biochemical markers were classified into one of the following four categories: useful for stratification of the disease, for monitoring of the disease, to assess response to treatment or to assess drug mode of action. As the current consensus paper did not aim to update the definition of sarcopenia, biochemical markers that only offer a diagnostic perspective of sarcopenia without any known physio-pathological explanation were not listed in the recommendations. Regarding analytical performances, to be selected for recommendations, the biomarkers should be measurable in an accurate and reproducible manner with a widely available method. The blood matrix was preferred over other matrices. Additional but not mandatory criteria were the existence of a standardized method, suitability for high-throughput analysis, feasibility of preanalytical conditions, existence of literature defining biological variation (available through https://biologicalvariation.eu) and other technical limitations.

### Selection of Biochemical Markers

The group has identified two sets of biochemical markers to be assessed in phase II or phase III pharmacological trials on sarcopenia. The first category of biochemical markers evaluating musculoskeletal status comprises biochemical markers of muscle mass, neuro-muscular junction, muscle turnover and myokines. The second set comprises biochemical markers evaluating nonmuscle-specific pathophysiological mechanisms, also referred to hereafter as causal factors. This second set includes three subclasses: adipokines, hormones, and inflammatory biochemical markers. At least one biochemical marker per subclass for each set, except for muscle mass biochemical markers (*see related section*), should be selected if the pharmacological trial is a phase II or a phase III trial. Recommendations for the selection of these chemical biochemical markers, as well as their time point assessment, are summarized in Table 1.

**Table 1:** Recommended biochemical markers for the conduct of any new phase II or phase III pharmacological trial in sarcopenia

	Phase II		Phase III	
	Recommendation	Time point assessment	Recommendation	Time point assessment
Assessment of musculoskeletal status				
Muscle mass				
D3creatine dilution test	N		N	
Myokines				
Myostatin – Follistatin	M	Every 3 months	M*	Every 3 months
Activin A	FRN		FRN	
GDF-15	O	Baseline	O	Baseline
Irisin	FRN		FRN	
Neuro-muscular junction				
CAF	N		N	
BDNF	M	Every 3 months	M	Every 3 months
Others				
PIIINP	M	Every 3 months	M	Every 3 months
Sarcopenia index	M	Baseline	M	Baseline
Assessment of causal factors				
Adipokines				
Adiponectin	O*	Every 3 months	O*	Every 6 months
Leptin	O*	Every 3 months	O*	Every 6 months
Hormones				
IGF-1	M	Every 3 months	M	Every 6 months
DHEAS	M*	Every 3 months	O*	Every 6 months
Cortisol	M*	Every 3 months	O*	Every 6 months
Testosterone	O*	Every 3 months	O*	Every 6 months
Inflammatory markers				
CRP	M	Every 3 months	M	Every 6 months
IL-6	M	Every 3 months	O	Every 6 months
TNF-α	M	Every 3 months	O	Every 6 months

*GDF-15* growth factor differentiation-15; *CAF* C-terminal agrin fragment; *BDNF* Brain derived neurotrophic factor; *PIIINP* N-terminal type 3 procollagen; *IGF-1* Insulin-like growth factor-1; *DHEAS* dehydroepiandrosterone sulphate; *CRP* C-reactive protein; *IL-6* interleukin-6; *TNF-α* tumor necrosis factor-α

The group agreed on four levels of recommendations based on the analytical and clinical properties: Mandatory biochemical markers that should be assessed in any new trial (M); Optional biochemical markers that may bring interesting mechanistic insights depending on the mode of action of the pharmacological therapy (*O*); Not recommended biochemical markers due to analytical limitations (*N*); Biochemical markers requiring further research needs and not recommended for the moment (FRN)

*Specific recommendations are applicable *(see related section)*

### Set 1. Musculoskeletal biochemical markers

This first set of biochemical markers is intended to evaluate the musculoskeletal status of sarcopenic patients. Ideally, biochemical markers included in this set should be highly specific to muscle, associated with muscle mass or strength, and sensitive to interventional trials. Additionally, the previous “ESCEO update on recommendations for the conduct of clinical trials for drugs aiming at the treatment of sarcopenia” identified several biochemical markers of “muscle-bone interaction” as potentially applicable outcomes for phase II studies [7]. Major clinical evidence and analytical constraints for using this set of musculoskeletal biochemical markers in pharmacological trials are reported in Tables 2 and 3.

**Table 2:** Clinical evidence for the use of musculoskeletal biochemical markers in the conduct of pharmacological trials in sarcopenia

Assessment of musculoskeletal status	Modification in sarcopenia	Evidence	Weakness	Associated disease	Mechanism
Muscle mass					
D3creatine dilution test	Decreased	Direct measurement of muscle mass [15]	Most data on the same male cohort [155]	Unknown	Exogenous biomarker
		Associated with physical performance, handgrip strength but not systematically with walking speed [16, 156]	Rare studies in female [155]		
		Predictive of major outcome (Falls, fracture, mobility, mortality) [18–20]	Longitudinal studies only available in rats [157]		
Myokines					
Myostatin	sex dependent	Increased with higher muscle mass [26, 158, 159]	Conflicting data [25]	Decreased upon inflammation [26, 160]	Muscle protein turnover
		Gender dependent association with gait speed [161]		Increased with obesity and heart failure [23, 162]	
		Probably decreased in long-term training [33]		Increased with renal insufficiency [26, 158]	
Follistatin	Increased	Associated with muscle mass and function in women [28, 31]	Conflicting data between men and women [28]	Unknown	Muscle protein turnover
		Increased in long-term training [33]			
Activin A	Unknown	Target same receptors as myostatin [163]	Limited number of studies [163]	Unknown	Muscle protein turnover
GDF-15	Increased	Correlate with muscle mass and performance tests [42–44]	Limited number of studies [163]	Increased with renal insufficiency [49]	Muscle protein turnover
		Not modified by exercise in long-term interventional studies [32, 35]			
Irisin	Decreased	Associated with muscle mass and strength [56, 164]	No interventional studies [80]	Unknown	Muscle protein turnover
		Increased by exercise [38]			
Neuro-muscular junction					
CAF	Increased	Associated with muscle mass and handgrip strength [58–62]	Limited number of interventional studies [80]	Unknown	Remodelling of neuro-muscular junction
		Decreased with exercise [63, 165]			
BDNF	Decreased	Accurate for diagnosis of sarcopenia [68, 69] Correlate with CAF [68, 69]	Limited number of studies [80]	Unknown	Remodelling of neuro-muscular junction
Others					
PIIINP	Probably decreased	Correlate with muscle mass [76–79]	Conflicting data between men and women [78, 84]	Unknown	Muscle collagen turnover
		Increased by testosterone administration [81, 82]			
		Predictive of mobility decline [79]			
Sarcopenia index	Decreased	Correlate with muscle mass, handgrip strength and gait speed [85–87]	Studies in chronic disease cohorts rather than normal aging [85–87]	Probably not efficient in acute kidney disease [166]	Developed to reflect muscle mass
		Correlate with malnutrition [89]	No interventional studies [166]		
		Highly predictive of major outcome (mortality and hospitalization) [85, 86, 91–94]			

*GDF-15* growth factor differentiation-15; *CAF* C-terminal agrin fragment; *BDNF* brain derived neurotrophic factor; *PIIINP* *N*-terminal type 3 procollagen

**Table 3:** Analytical aspects for the use of biochemical markers for the conduct of pharmacological trial in sarcopenia

	Type of assay	Matrix	Automatization	Widely available	Preanalytical constrains*	Biological variation	Limitations
Assessment of musculoskeletal status							
Muscle mass							
D3creatine dilution test	LC–MS/MS	Urine	None	No	Fasting for morning urine	Unknown	Need a correction based on creatinine
Myokines							
Myostatin	LC–MS/MS	Serum	None	Yes	24 h exercise-free period	Sex dependent	
	ELISA	Plasma				Uncertainties about age-dependent levels	ELISA specificity is controversial
Follistatin	ELISA	Serum Plasma	None	Yes	24 h exercise-free period	Sex dependent	
Activin A	ELISA	Serum Plasma	None	Yes	24 h exercise-free period	Uncertainties about age and sex-dependent levels	
GDF-15	ELISA	Serum Plasma	None	Yes	24 h exercise-free period	Age dependent	
Irisin	ELISA	Serum Plasma	None	Yes	Unknown	Unknown	
Neuro-muscular junction							
CAF	ELISA	Serum	None	No	Unknown	Uncertainties about sex-dependent levels	Kits unavailable
BDNF	ELISA	Serum Plasma	None	Yes	24 h exercise-free period	Age and sex dependent	
Others							
PIIINP	RIA ELISA ECLIA	Serum	Only with ECLIA	Yes	Fasting	Unknown	High variabilities between techniques
Sarcopenia index	Colorimetric Enzymatic Turbidimetric	Serum Plasma	Yes	Yes	No preanalytical constrains	Based on data for creatinine and cystatin	Only the non eGFR based formula is recommended
Assessment of causal factors							
Adipokines							
Adiponectin	ELISA ECLIA	Serum Plasma	Only with ECLIA	Yes	Fasting	Age, BMI and sex dependent	
						BV described	
Leptin	ELISA	Serum Plasma	None	Yes	Fasting	Age, BMI and sex dependent	
Hormones							
IGF-1	ECLIA	Serum Plasma	Yes	Yes	No preanalytical constrains	Age and sex dependent	
DHEAS	LC–MS/MS ECLIA	Serum Plasma	Yes	Yes	No preanalytical constrains	Age and sex dependent	
						BV described	
Cortisol	LC–MS/MS ECLIA	Serum Plasma Saliva Urine	Only with ECLIA	Yes	Standardize time of collection for blood	Circadian rhythm BV described	Prefer use of total rather than free hormone Prefer use of serum/plasma
Testosterone	LC–MS/MS ECLIA	Serum Plasma	Yes	Yes	Fasting Morning collection (between 7 and 10 AM)	Age and sex dependent BV described	Prefer use of total rather than free hormone
Inflammatory markers							
CRP	Turbidimetric	Serum Plasma	Yes	Yes	24 h exercise-free period	BV described	Prefer use of ultrasensitive method
IL-6	ELISA Cytokine panel ECLIA	Serum Plasma	Yes	Yes	24 h exercise-free period	BV described	
TNF-α	ELISA Cytokine panel	Serum Plasma	Yes	Yes	24 h exercise-free period	BV described	

*GDF-15* growth factor differentiation-15; *CAF* C-terminal agrin fragment; *BDNF* brain-derived neurotrophic factor; *PIIINP* *N*-terminal type 3 procollagen; *IGF-1* Insulin-like growth factor-1; *DHEAS* dehydroepiandrosterone sulphate; *CRP* C-reactive protein; *IL-6* interleukin-6; *TNF-α* tumor necrosis factor-α; *LC–MS/MS* Liquid chromatography and tandem mass spectrometry; *ELISA* enzyme-linked immunosorbent assay; *ECLIA* electrochemiluminescence immune assay; *BV* biological variation

* Fasting or 24 h exercise-free period are optional constrains when not mentioned

### Biochemical Markers Specific to Muscle Mass

In addition to imaging techniques, a few biochemical tools, namely, the deuterium-labelled creatine (D3-Cr) dilution test and sarcopenia index (SI), were developed to evaluate muscle mass. In pharmacological trials, biochemical markers specific to muscle mass are expected to provide additional information that may help in patient risk stratification. Of note, given the poor specificity of SI to muscle mass, this ratio has been reclassified in the muscle turnover subclass.

*The D3 creatine dilution test* has been developed to ensure an analytical quantification of muscle mass, where imaging techniques are more considered to reflect fat-free mass or bone- and fat-free lean mass [15]. Thus, although well correlated, dual-energy *X*-ray absorptiometry (DXA) and D3-Cr dilution tests should not be considered equivalent [16]. The D3-Cr dilution test is based on the ingestion of an oral solution of deuterium-labelled creatine (D3-Cr) by a fasting patient followed by the measurement of both the labelled and total creatine and creatinine by liquid chromatography coupled with tandem mass spectrometry (LC‒MS/MS) in urine before and four days after ingestion [17]. As creatine directly enters the muscles where a certain amount is nonenzymatically converted to creatinine to be excreted in urine, an algorithm based on the ratio of D3-Cr to unlabelled creatine can provide an accurate measurement of muscle mass.

The D3-Cr dilution test has shown interesting clinical evidence, such as associations with “hard” clinical outcomes. Indeed, D3-Cr muscle mass appears to be an independent predictor of self-reported incident mobility disability together with walking speed [18] but also a predictor of self-reported disabilities in activities of daily living [19] and risk of hip fracture [20]. However, most of these observations have been made in the same cohort of older men (the osteoporotic fracture in men cohort) [18–20], and the D3-Cr dilution test was only evaluated in one cohort of postmenopausal women [21]. Furthermore, most often D3-Cr muscle mass was divided by body weight in the abovementioned studies, whereas body weight itself is associated with these difficult clinical outcomes.

From an analytical perspective, this method has some limitations that make the D3-Cr dilution test impractical for use in pharmacological trials. Indeed, it is only available in a few highly specialized laboratories without any external quality assessment or ring-test that allows lab-to-lab data comparison. Of note, this method requires a standardized procedure for D3-Cr ingestion and urine collection.

**Table:** 

Given the analytical limitations of the D3-Cr dilution test, the group recommends not using the method in pharmacological trials at present. In addition to the need for a more widely available method, the group also identified that the method should be validated in other cohorts before any recommendation in clinical trials. Therefore, also taking into account that it is presently the only analytical test highly specific to muscle mass, the group concluded that muscle mass should be assessed according to the EWGSOP2 revised definition without additional biochemical markers

### Myokines

Myokines are muscle-secreted small proteins (5–20 kDa) with autocrine, paracrine or endocrine effects. Myokines contribute to muscle maintenance, acting, for example, on metabolism, angiogenesis, and inflammation [22]. In aging, myokine secretion and the sensitization of the muscle to these myokines are altered, leading to a disturbance in the balance between anabolic and catabolic effects with consequent age-related muscle atrophy [23]. Therefore, the group agreed that myokines are interesting tools in pharmacological trials to decipher the drug mode of action and physio-pathological mechanisms of muscle protein turnover. Myokines might also be helpful in evaluating secondary outcomes for phase II studies. However, it is premature to determine whether myokines are useful for patient stratification, monitoring of therapies or monitoring side effects.

*Myostatin, also called growth and differentiation factor 8 (GDF-8),* is a member of the transforming growth factor-*β* (TGF-*β*) superfamily and is mostly seen as a muscle growth suppressor [22]. Indeed, when binding activin type IIA and IIB receptors or TGF-*β* receptors, myostatin suppresses mammalian target of rapamycin (mTOR)-mediated protein synthesis [24]. However, some argue that myostatin can promote muscle growth through different downstream pathways [22]. Myostatin is considered specific to skeletal muscle even if it is also expressed in adipose and cardiac tissues [22].

Myostatin is probably the most studied myokine. However, many studies have yielded conflicting data on the relationship between myostatin and its role in age-related muscle atrophy. Indeed, while most studies found an association between higher myostatin blood concentration and higher muscle mass, this association is not systematically observed [25]. Nevertheless, myostatin is a good predictor of one-year mortality as a “hard” clinical outcome in patients on hemodialysis [26]. Regarding the association between myostatin and muscle function, studies have shown either increased myostatin concentrations in participants with better muscle function or no (or only men-specific) association [25]. Part of these conflicting data may stem from a sex-dependent expression pattern [27, 28]. Regarding its variability with age, myostatin assessment through a highly specific LC‒MS/MS method showed a decline in myostatin concentration with aging in men but not in women [29]. However, many other studies have shown a relatively steady state or increased serum myostatin level with age [25, 30].

Another part of these discrepancies may come from the analytical heterogeneity of the assays. Indeed, myostatin is highly similar to its homologous growth and differentiation factor 11 (GDF-11) [29]. Thus, older kits are known to present significant cross-reactivity with several TGF-β superfamily members [27]. Therefore, whenever possible, LC‒MS/MS methods to measure myostatin should be favoured.

*Follistatin* is an antagonist of TGF-*β* ligands, including myostatin and activin A, by acting on myogenic transcription factors. Follistatin has been correlated with muscle mass and muscle function in women in a small number of studies [28, 31], but this association was not confirmed in men [28]. However, in mid- to long-term resistance training intervention trials, follistatin and/or follistatin/myostatin ratio were increased. The same observation was not consistently found for myostatin alone [32–35]. Analytically, several ELISA and RIA assays are easily available to measure follistatin, but preanalytical considerations and biological variability are not properly described [36].

The myostatin-follistatin system has long been considered a possible target for sarcopenia therapies, and several clinical trials are ongoing, as reviewed by Skrzypczak and colleagues [37]. Although it is unclear whether these proteins are good biochemical markers to monitor the disease or the drug efficacy, myostatin and follistatin should be considered as a couple that helps decipher the physio-pathological drug mode of action.

**Table:** 

The group recommends that clinical trials with drugs directly targeting the myostatin/follistatin system should follow the two proteins in both phase II and phase III trials with a precise statistical analysis for men and women. To this end, the follistatin/myostatin ratio can be optionally calculated. However, when the target is an entirely different system, these biochemical markers should be measured in phase II and considered optional in phase III if no changes have been observed in the phase II study. Additionally, when physical training is associated with pharmacological therapies, the standardized procedure for blood collection should include a delay of at least 24 h between the last exercise and venepuncture. Indeed, myostatin and follistatin are acutely increased several hours after exercise but return to baseline after approximately 24 h [38]

*Activin A* is another member of the TGF-*β* superfamily that preferentially binds the activin type IIA receptor. Activin A is also considered a negative regulator of muscle growth, acting through the same pathways as myostatin. However, Activin A’s contribution to sarcopenia physiopathology is still a theoretical concept with an evident lack of cohort-based evidence.

**Table:** 

The group considers that future research is required before any recommendation for using Activin A in clinical trials for sarcopenia can be made

*Growth factor differentiation-15 (GDF-15)* is also a member of the TGF-β superfamily, and its expression is induced by stress or myocardial infarction [39]. Several cohorts have shown increased GDF-15 expression in sarcopenic participants [40, 41]. GDF-15 was also sometimes, but not systematically, associated with handgrip strength, skeletal muscle index (SMI) [42–44] and physical performance tests [45, 46]. Myostatin and follistatin expression is induced upon acute physical exercise [47]. However, the few long-term interventional trials that tested GDF-15 expression upon resistance training in a sarcopenic cohort failed to observe any longitudinal change in GDF-15 expression [32, 35]. The GDF-15 could also not predict sarcopenia occurrence or evolution in a 2-year follow-up period [40, 48].

Several ELISAs are available on the market for GDF-15 measurement. Nevertheless, GDF-15 is increased with age and chronic kidney disease [49], and doubts have been raised regarding the existence of a circadian rhythm [39]. Given these analytical and clinical considerations, GDF-15 does not appear to be a good biomarker to assess response to treatment. However, adding GDF-15 concentrations to physical performance tests may have some added value to understanding the therapies' effects on physio-pathological mechanisms.

**Table:** 

Given that GDF-15 has shown some interesting associations with muscle mass, muscle strength and performance tests but has failed thus far to show modifications in longitudinal follow-up, the group recommends the optional use of GDF-15 in phase II or phase III considering that baseline levels may help in stratifying patients. The standardized measurement procedure should include a 24 h free exercise period before venepuncture and a defined time for blood collection

*Irisin* is a myokine secreted by skeletal muscles under physical exercise, although it is not a member of the TGF-*β* superfamily. Irisin is responsible for the browning of white adipose tissue [50], and irisin injection in mice induces muscle hypertrophy with increased protein synthesis [51]. This mechanism might be at least partially regulated by the myostatin/follistatin couple, as irisin is increased in myostatin knock-out mice or after recombinant follistatin injection [50].

Irisin is decreased in sarcopenic persons in several cohorts with very few discordant data [52–54]. Irisin is also regularly associated with muscle strength or mass [55, 56]. Nevertheless, irisin has not been studied thus far either in non-pharmacological interventional trials or as a prognostic biomarker of outcomes. Analytically, the analytical precision of ELISA is not accurate enough, and physiological variation is poorly characterized [57].

**Table:** 

The group considers it premature to recommend irisin use in clinical trials at present

### Neuromuscular Junction

By including performance tests and muscle strength in the definition of sarcopenia, the EWGSOP group states that sarcopenia is “a multidimensional concept that not only involves muscles but also central and peripheral nervous function, including balance” [1]. Therefore, not only muscle-specific biochemical markers but also biochemical markers of the neuromuscular junction are required to evaluate muscle integrity, as any impairment to the neuromuscular junction could lead to decreased capacities in using muscles and producing volitional tasks. In pharmacological trials, any modification of these biochemical markers should be integrated with performance tests to understand potential drug modes of action. It is premature to determine whether these biochemical markers may help monitor the disease. However, it is conceivable that these biochemical markers could reflect positive effects on cognition or neurological side effects of therapies.

*C-terminal agrin fragment (CAF)*, also called CAF-22 when referring to the smaller fragment of 22 kDa, is a byproduct of agrin released during the remodeling of the neuromuscular junction. CAF is increased in sarcopenic patients in various cohorts and is associated with the skeletal muscle index [58–62]. It has also been reported to be lower in older dancers than in their sedentary counterparts [63]. Clinically, this biomarker looks very promising. However, there is currently no commercially available assay for CAF.

**Table:** 

Given that the appropriate technology is not available at the moment, the group does not currently recommend this biomarker in clinical trials. Nevertheless, as a clinically promising biomarker, developing a widely available and accurate method for CAF determination could modify this recommendation

*Brain-derived neurotrophic factor (BDNF) and glial cell line-derived neurotrophic factors (GDNF)* are other biochemical markers of neuromuscular junctions and neuroinflammation. BDNF and GDNF are neurotrophic factors expressed by motor neurons [64]. BDNF and GDNF participate in motor axonal regeneration and neuronal plasticity through a paracrine effect [65]. BDNF and GDNF are lower in sarcopenia of hemodialyzed or kidney transplant patients [66, 67]. Both factors were shown to be as efficient as CAF for the diagnosis of sarcopenia in chronic obstructive pulmonary disease patients [68] and in Parkinson’s disease patients [69]. However, BDNF has been studied more in sarcopenia than GDNF.

BDNF is measurable through various ELISA kits. BDNF expression is controlled by sex hormones [70], and as a neuronal biomarker, BDNF expression is modified in many psychiatric disorders [71]. Thus, all these confounding factors should be included in the statistical analysis. Additionally, BDNF is increased upon acute exercise, but its level after long-term training still has to be determined [72, 73].

**Table:** 

The group recommends BDNF measurement at baseline and follow-up in phase II and phase III studies to evaluate the neuromuscular part of the disease. For other biochemical markers that increase upon acute exercise, venepuncture should be performed at least 24 h after the last acute physical exercise

### Other Biochemical Markers of Muscle Turnover

*N-terminal type III procollagen (PIIINP)* is a byproduct of the synthesis of type 3 collagen. Type III collagen is expressed in smooth muscles and in the endomysium of skeletal muscle to enhance tissue stretching properties [74, 75]. In sarcopenia, PIIINP has been associated with the skeletal muscle index and, to a lesser extent, physical performance but not muscle strength in several distinct cohorts [76–79]. Additionally, PIIINP is a biomarker of choice to measure muscle remodeling, as it reflects an anabolic response compared to steroid hormones, which are indicators of hormonal status [80]. Indeed, it appears to be a good biomarker of anabolic response to therapies with testosterone or growth hormone [81, 82]. PIIINP also shows a small-to-moderate increase in interventional trials on small cohorts of older participants [83, 84].

Analytically, PIIINP is a serum biomarker measurable through several different techniques (ELISA, RIA, ECLIA). Additionally, although not described, preanalytical constraints are expected to be the same as for N-terminal type I procollagen (PINP).

**Table:** 

The group recommends measuring PIIINP as a follow-up biomarker in phase II and phase III studies to evaluate overall muscle turnover. Sampling should be realized in fasting individuals as a precaution until further studies are assessing the preanalytical questions

The serum creatinine to serum cystatin C ratio, or *sarcopenia index* (SI), is a recent index first created to evaluate muscle mass [85]. Indeed, serum creatinine can be seen as a biomarker of muscle protein turnover [80]. However, its blood concentration is highly dependent on renal function. Thus, this ratio was developed to circumvent this limitation by normalizing creatinine with cystatin C, another biomarker of renal function. SI has shown a moderate correlation with CT muscle cross-sectional area, calf circumference and handgrip strength [85–87]. Nevertheless, SI is not sensitive and specific enough to be used as a biomarker of muscle mass [88]. However, SI has been shown to be associated with malnutrition in critically ill patients [89] and in patients suffering from cirrhosis [90]. Additionally, it was associated with clinical outcomes and especially hospitalization and/or mortality in hospitalized older patients [86], critically ill patients [85], cardiac patients [91, 92] and cancer patients [93, 94]. Thus, it is important to consider SI as a way of stratifying the risk of outcomes rather than a real muscle mass assessment. Given the lack of interventional studies thus far, its usefulness as a treatment follow-up is unclear.

Analytically, this index is easy to calculate from well-described biochemical markers in terms of physio-pathological and analytical variability, with widely available high-throughput methods. However, the use of an enzymatic method for creatinine should be preferred compared to a Jaffe method. Indeed, the enzymatic method is analytically more sensitive and specific than the Jaffe method [95]. Additionally, the creatinine assay should be traceable to isotope dilution mass spectrometry (IDMS), and cystatin C assays should be traceable to the ERM-DA471/IFCC reference material to ensure the consistency of the results.

Two formulas for SI coexist in the literature. The first one is the original one defined as [serum creatinine (mg/dL)/serum cystatin C (mg/L)] × 100. The second one emerged a few years later and is calculated as the serum creatinine X cystatin C-based glomerular filtration rate (eGFR_cysC_) [96]. Although this second formula has shown better correlation with muscle mass and handgrip strength [96, 97], we do not recommend its use in multifactorial statistical approaches. Indeed, eGFR_cysC_ is calculated using the Chronic Kidney Disease Epidemiology Collaboration equation, which contains age and gender as variables [98]. Therefore, the use of this second formula introduces potential confounding factors in the index rather than as covariates in the statistical models.

**Table:** 

The group recommends the use of the SI index with the formula (serum creatinine (mg/dL)/serum cystatin C (mg/L)) X 100 at least at baseline in both phase II and phase III studies to help in patient risk stratification. Longitudinal research is needed to define SI added value during follow-up

### Set 2 Causal factor evaluation

As a multifactorial disease, sarcopenia-related loss of muscle mass and strength is related not only to muscle metabolism dysfunction but also to metabolic and endocrine abnormalities, including chronic low-grade inflammation. Thus, it is important to follow not only muscle-specific biochemical markers but also biochemical markers that reflect the patient’s metabolic, endocrine and inflammation status. Ideally, biochemical markers included in this set should be modified in sarcopenic persons compared to healthy controls, and their normalization should be considered beneficial for the patient. In pharmacological trials, measurement at baseline may help in patient risk stratification. Additionally, the improvement in these biochemical markers might be considered a secondary outcome in phase II and phase III studies. Major clinical evidence and analytical constraints for assessing these causal biochemical markers in pharmacological trials are reported in Tables 3 and 4.

**Table 4:** Clinical evidence for the use of causal biochemical markers of sarcopenia in the conduct of pharmacological trials in sarcopenia

Assessment of causal factors	Modification in sarcopenia	Evidence	Weakness	Associated disease	Mechanism
Adipokines					
Adiponectin	Increased	Associated with physical performance tests [167]	Results are influenced by the ratio male/female [113]	Pro-inflammatory factor [168]	Muscle-fat crosstalk
		Modified by non-pharmacological interventions [106, 169]	Conflicting data [107]	Decreased in coronary heart disease [168]	
		Predictive of major outcome (all-cause mortality) [110]		Decreased in obesity and type 2 diabetes [168]	
Leptin	Increased	Associated with appendicular lean mass [103, 106, 115]	Studies in sarcopenic obesity and insulin resistance cohorts rather than normal aging [103, 116, 119]	Influenced by level of adiposity [23]	Muscle-fat crosstalk
		Modified by non-pharmacological interventions [106, 116]	Highly relevant in sarcopenic obesity [80]		
Hormones					
IGF-1	Decreased	Associated with muscle mass, handgrip strength and gait speed [30, 125]	Not accurate enough for diagnosis [122]	Decreased with hepatic disorder [170]	Anabolic
		Increased by long-term resistance training [126]			
DHEAS	Decreased	DHEAS treatment improve muscle strength and function when combined with exercise [134]	Modified secretion is part of aging [171]	Modified by diseases of the endocrine system [171]	Anabolic
		Modified by non-pharmacological interventions [133]			
Cortisol	Increased	Associate with muscle mass [130, 172]	Modified secretion is part of aging [171]	Modified by diseases of the endocrine system [171]	Anabolic
		Modified by non-pharmacological interventions [133, 135]			
Testosterone	Probably decreased	Testosterone treatment improve muscle strength and mass [134]	Mostly studied as pharmacological target rather than biomarker [134, 135]	Modified by diseases of the endocrine system [171]	Anabolic
Inflammatory markers					
CRP	Increased	Increase is associated with lower handgrip strength and muscle mass [139, 172]	Unspecific biomarker of frailty and aging [155]	Increased by chronic or acute inflammation [140]	Low grade chronic inflammation
		Associated with future decline of muscle strength [141, 172]			
		Modified by non-pharmacological interventions [135, 144]			
IL-6	No basal modification	Increase is associated with lower handgrip strength and muscle mass [139]	Unspecific biomarker of frailty and aging [155]	Increased by chronic or acute inflammation [140]	Low grade chronic inflammation
		Associated with future decline of muscle strength [141]			
TNF-α	No basal modification	Increase is associated with lower handgrip strength and muscle mass [139]	Unspecific biomarker of frailty and aging [155]	Increased by chronic or acute inflammation [140]	Low grade chronic inflammation
		Modified by non-pharmacological interventions [135]			

*IGF-1* Insulin-like growth factor-1; *DHEAS* dehydroepiandrosterone sulphate; *CRP* C-reactive protein; *IL-6* interleukin-6; *TNF-α* tumor necrosis factor-α

### Adipokines

Adipokines are adipocyte-secreted proteins involved in insulin resistance, glucose consumption by the muscle, lipolysis and inflammatory processes [99]. Muscle-fat cross talk is now well established, and measurement of adipokines partially evaluates metabolism abnormalities occurring in sarcopenia [23]. In pharmacological trials, the group agreed that adipokines are particularly relevant when therapies involve changes in body fat mass or insulin resistance to help decipher molecular modes of action. However, it is unclear whether adipokines are useful for patient risk stratification, monitoring of therapies or side effects.

*Adiponectin* is an adipokine involved in insulin resistance and inflammatory processes. The inflammatory function is partially achieved through the synthesis of cytokines such as interleukin-6 (IL-6) and tumor necrosis factor α (TNF-*α*) [100], while adiponectin-mediated insulin resistance occurs through the reduction of glucose uptake by modulating the interaction between adiponectin receptor AdopiR1 and adaptor protein containing pleckstrin homology domain (APPL1) [101]. In mice, adiponectin was shown to induce muscle regeneration by binding to *T*-cadherin [102].

Adiponectin has been inversely associated with appendicular lean mass [103, 104]. However, when body composition is considered a cofounding factor, this association disappears [103, 104]. Additionally, conflicting data coexist regarding the level of adiponectin in sarcopenic persons [103, 105, 106]. However, a recent meta-analysis concluded an increased level in sarcopenia regardless of body fat levels [107]. This finding is in line with the adiponectin paradox. In centenarians, adiponectin is higher and positively correlated with biochemical markers of low cardiovascular risk, such as HDL or reduced insulin resistance [108, 109]. However, in people > 65 years old, high levels of adiponectin are frequently associated with insulin resistance, metabolic disorders or mortality [110, 111]. Nevertheless, long-term physical interventions may increase adiponectin with a beneficial impact on muscle [23, 112]. Komici et al. have demonstrated that adiponectin increase in sarcopenia might represent a compensatory effect solicited by chronic inflammation and oxidative stress [107].

Analytically, adiponectin is easy to measure, and some automated methods are available. The physiological variations are well described and include age, BMI, fat mass and sex [113].

**Table:** 

The group recommends the optional use of adiponectin during both phase II and phase III studies at baseline and during the whole follow-up to evaluate the contributing processes linking adipose tissue and sarcopenia. Changes in BMI, muscle density and any body composition measure should absolutely be integrated in the statistical analysis, as these changes can confound the association between adiponectin and sarcopenia factors [104]

*Leptin* is a pro-inflammatory adipokine that is often considered to have antagonistic effects on adiponectin, although it acts through different pathways [99]. By causing inflammation, exogenous leptin can cause muscle atrophy through protein synthesis reduction in myocytes [80]. However, inflammatory cytokines often do not correlate with leptin levels [114]. Leptin is higher in sarcopenic persons and often associated with lower appendicular lean mass [103, 106, 115]. However, for adiponectin, the strength of this association is reduced when fat mass is considered a cofounding factor [103]. Interestingly, leptin was reduced after exercise combined with nutritional intervention or exercise alone in a randomized controlled trial [116]. In this study, although not significant, leptin reduction tended to be smaller in the group with a smaller change in body fat mass.

Analytically, leptin can be measured by ELISA, but biological variation is not described. Nevertheless, leptin levels are higher in women even after adjustment for fat mass and decrease with age [117]. Obesity is also well known to influence leptin levels [118]. Hence, leptin is higher in sarcopenic obese patients than in normal sarcopenic hemodialyzed patients [119].

**Table:** 

The group agrees that at least adiponectin or leptin should be measured in both phase II and phase III trials at baseline and during the whole follow-up. Nevertheless, because leptin appears to give more consistent results across the literature compared to adiponectin, leptin should be preferred to investigate the crosstalk between adipose tissue and muscle. The adiponectin-leptin ratio is another acceptable option. For both adiponectin and leptin, cofounding factors such as BMI, muscle density and any change to body composition or body fat mass should be adjusted for in the statistical analysis [23]

### Hormones

*Insulin-like growth factor-1 (IGF-1)* is sometimes considered a growth factor or sometimes a myokine of low specificity to the muscle [22]. Nevertheless, IGF-1 has growth hormone (GH)-mediated anabolic properties, notably upon physical exercise [120]. Thus, IGF-1 behaviour in sarcopenia has been largely studied, and IGF-1 age-related decline is considered to be a causal factor of the disease [121]. Alone, IGF-1 specificity for the diagnosis of sarcopenia is low, although IGF-1 has shown promising features in diagnosing sarcopenia when combined with a panel of biochemical markers [122]. Nevertheless, studies have shown that IGF-1 is lower in sarcopenic persons [122–124], correlates with muscle mass, hand grip strength, and gait speed [30, 125] and is increased upon long-term resistance training [126].

Analytically, the total IGF-1 or IGF-1 bioactive form can be measured. Total IGF-1 is easily measurable through largely available automated methods, and thus, total measurement should be favoured. The major confounding factor that must be considered is age, as IGF-1 is well described to decrease with age. Importantly, the dosage of GH cannot be used to evaluate the GH/IGF-1 axis. Indeed, due to the pulsatile secretion of GH, the intraindividual biological variation of GH is high [127], and there are no preanalytical procedures that may reduce this variability.

**Table:** 

The group recommends only using IGF-1 to study the GH/IGF-1 axis. Its measurement should be realized in both phase II and phase III trials at baseline and follow-up

*Steroid hormones* have long been studied in sarcopenia. Dehydroepiandrosterone sulphate (DHEAS) and testosterone age-related decline are often considered causal factors for muscle loss, as the aging phenotype shares common features with hypogonadism in younger men [128]. Cortisol, unlike testosterone and DHEAS, displays well-known catabolic properties, and higher cortisol levels are associated with frailty or sarcopenia [129, 130]. Nevertheless, as a biomarker of sarcopenia, DHEAS is the most studied steroid hormone. DHEAS is decreased in sarcopenia, while cortisol is increased, probably due to chronic inflammation [131, 132]. Both DHEAS and cortisol blood levels have been shown to be modified by nutritional or nutritional-exercise interventional trials [133]. Although testosterone has been tested as a drug in a wide variety of clinical trials, its usefulness as a biomarker is mainly based on concepts rather than cohort-based evidence [134, 135].

Analytically, the three parameters are widely measurable through automated methods. Obviously, as a steroid hormone, these parameters are age- and gender dependent. The circadian rhythm of cortisol includes a nadir at midnight and a morning peak [136]. A ratio between DHEAS and cortisol has also been proposed [131]. However, further data are needed before any recommendation can be made on this ratio.

**Table:** 

The group considers that DHEAS and cortisol should be monitored in phase II pharmacological trials. In phase III trials, the use of these biochemical markers should be regarded as mandatory in case any changes have been observed during phase II trials. Otherwise, DHEAS and cortisol are optional biochemical markers for phase III trials. Given that DHEAS is a testosterone precursor, the group recommends using testosterone only in trials where testosterone is part of the therapy mechanism. For the three parameters, blood compared to salivary and total hormone compared to free hormone should be favoured. For cortisol, the time of collection should be standardized

### Inflammatory Biochemical Markers

Chronic inflammation has long been described as a fundamental mechanism in sarcopenia [137]. Many cytokines have been proposed to be dysregulated in sarcopenia, and a “core cytokinome” in sarcopenia and frail persons has been published [138]. Based on the investigation of 27 inflammatory molecules, the authors showed that C-reactive protein (CRP) is increased, while myeloperoxidase, interleukin-8 (IL-8), monocyte chemoattractant protein-1 and platelet-derived growth factor BB are decreased. Additionally, a recent meta-analysis found that among 168 articles studying the links between inflammatory molecules and muscle strength or mass, the three more studied inflammatory molecules are CRP, IL-6 and TNF-*α* [139].

*CRP, IL-6, and TNF-α* have been associated with a decline in muscle mass and muscle strength in a meta-analysis [139]. Nevertheless, in a second meta-analysis, CRP, but not IL-6 or TNF-*α*, was shown to be increased in sarcopenia [140]. Moreover, higher CRP and IL-6 are associated with a future decline in muscle strength [141]. Additionally, several nutritional intervention trials in older adults or in sarcopenic patients showed marked reduction of these biochemical markers after several weeks of intervention [135, 142]. Obviously, the nature of the intervention and the provided compounds are linked to the strength of the observed effect [143]. Furthermore, a meta-analysis on resistance training concluded a beneficial effect of resistance training on CRP but not on IL-6 and TNF-*α* [144], while acute training is known to show a short-term increase in these biomarkers [145]. Thus, clinically, CRP seems to be the most interesting biomarker of chronic inflammation in sarcopenia. Furthermore, analytically, methods for measuring CRP are the most automated and widely available compared to methods for IL-6 and TNF-α measurement.

**Table:** 

The group recommends at least the use of CRP at baseline and follow-up as a biochemical marker of chronic inflammation in both phase II and phase III clinical trials, whereas IL-6 and TNF-α should only be considered optional biochemical markers. Of note, only the ultrasensitive method for CRP measurement should be used because the “classical” method for CRP detection might not be sensitive enough to detect any clinically relevant changes

### Analytical Recommendations

Several analytical and preanalytical considerations should be followed to ensure the accuracy of the measurements. First, a standardized collection procedure per biomarker should be established, and the type of collection tube, time for collection, time for coagulation, need for a fasting or an exercise-free period, centrifugation process and decantation process should be defined. Major specific preanalytical constraints per biochemical marker are listed in Table 3. It is especially important to define whether fasting or a 24 h exercise-free period are needed. The storage procedure should also be standardized, and freeze‒thaw cycles should be avoided. Additionally, it is important to ensure a centralized measurement procedure of biochemical markers in a certified and experienced laboratory. The laboratory should subscribe to external quality evaluations whenever available. Regarding the validation procedure, all methods should be appropriately validated according to ISO 15189 guidelines. This validation should at least include an establishment of the coefficient of variation (CV) of the dosage through repeatability and reproducibility studies and the definition of the lower limit of quantification. Reference ranges should be checked as well. Whenever feasible, the method should be compared to another laboratory method allowing the measurement of the same biomarker. Regarding the measurement procedure, all the analyses should be performed in a batchwise manner to limit lot-to-lot variations. The staff should be adequately trained, and traceability ensured. Finally, statistical analysis should include careful adjustments for potential confounders, including physical activity level and body fat, when investigating the association between biochemical markers and muscle parameters, physical function or hard clinical endpoints.

### Future Research Needs

Overall, few meta-analyses are available in the literature regarding the biochemical markers for sarcopenia, and specific studies should be dedicated to this task. Additionally, two primary pathophysiological mechanisms in sarcopenia are uncovered by the recommended panel of biochemical markers: nutritional status and oxidative stress. For nutritional status, the “update on the ESCEO recommendation for the conduct of clinical trials for drugs aiming at the treatment of sarcopenia in older adults” recommends evaluating the nutritional status of all participants at inclusion and at least on each time point assessment using tools such as European Society of Clinical Nutrition and Metabolism (ESPEN) criteria or Global Leadership Initiative of Malnutrition (GLIM) criteria [7]. Because these criteria are free of biochemical markers, the present recommendation does not advise additional use of biochemical markers.

Many studies have been conducted on oxidative stress with inconsistent biomarker reporting [146–148]. A meta-analysis identifying the most studied and accurate biochemical markers of oxidative stress in sarcopenia is urgently needed. It is, therefore, premature to recommend some specific biochemical markers of oxidative stress to be monitored in sarcopenia.

Finally, several promising biomarkers with interesting clinical features have been identified. First, we already discussed the D3-Cr dilution test and CAF measurement for which clinical data are convincing, but we do not have the appropriate technology to ensure widely available and accurate measurement for pharmacological trials. Additionally, apelin [149] and fibroblast growth factor-21 (FGF-21) [150] as musculoskeletal markers, together with neurofilament light chains (NfL) for the neuromuscular junction [151], have been identified as promising biochemical markers with clinically relevant data but with a too restricted number of studies to recommend their use in clinical trials. Finally, in the future, innovative approaches such as miRNA panels [152] and microbiome analysis [153, 154] will probably find a place in the biochemical marker field, but it is certainly premature to define their clinical utility at the present time in sarcopenia.

## Conclusions

In this consensus report, experts from the ESCEO working group proposed a clear list of biochemical markers of musculoskeletal status and the pathophysiological mechanisms of sarcopenia that are recommended to be assessed in any phase II and phase III clinical trials of drugs aimed at the management of sarcopenia. Based on the analytical and clinical properties of biochemical markers, the group agreed on four mandatory biochemical markers evaluating musculoskeletal status that should be assessed in any new Phase II or Phase III trial, namely, the myostatin-follistatin couple, BDNF, PIIINP and the Sarcopenia Index [using the formula (serum creatinine (mg/dL)/serum cystatin C (mg/L)) ×100]. In addition, experts also agreed on six mandatory biochemical markers evaluating nonmuscle-specific physio-pathological mechanisms (i.e., causal factors) that should be assessed in any Phase II trials, namely, IGF-1, DHEAS, Cortisol, CRP, IL6, and TNF-*α*. IGF-1 and CRP are also recommended to be measured in any phase III trials. The recommendations made in this consensual report are based on the available evidence and the availability of proper methodologies for biomarker assessment. Further research and development of new methods of biochemical marker dosage may lead to the evolution of these recommendations.
