# Correction: Biochemical Markers of Musculoskeletal Health and Aging to be Assessed in Clinical Trials of Drugs Aiming at the Treatment of Sarcopenia: Consensus Paper from an Expert Group Meeting Organized by the European Society for Clinical and Economic Aspects of Osteoporosis, Osteoarthritis and Musculoskeletal Diseases (ESCEO) and the Centre Académique de Recherche et d'Expérimentation en Santé (CARES SPRL), Under the Auspices of the World Health Organization Collaborating Center for the Epidemiology of Musculoskeletal Conditions and Aging

**DOI:** 10.1007/s00223-023-01114-y

**Published:** 2023-07-24

**Authors:** Aurélie Ladang, Charlotte Beaudart, Jean-Yves Reginster, Nasser Al-Daghri, Olivier Bruyère, Nansa Burlet, Matteo Cesari, Antonio Cherubini, Mario Coelho da Silva, Cyrus Cooper, Alfonso J. Cruz-Jentoft, Francesco Landi, Andrea Laslop, Stefania Maggi, Ali Mobasheri, Sif Ormarsdottir, Régis Radermecker, Marjolein Visser, Maria Concepcion Prieto Yerro, René Rizzoli, Etienne Cavalier

**Affiliations:** 1 Department of Clinical Chemistry, CHU de Liège, University of Liège, Liège, Belgium; 2 Division of Public Health, Epidemiology and Health Economics, WHO Collaborating Center for Public Health Aspects of Musculo-Skeletal Health and Ageing,, University of Liège, Liège, Belgium; 3 Biochemistry Department, College of Science, Chair for Biomarkers of Chronic Diseases, King Saud University, Riyadh, 11451 Saudi Arabia; 4 Department of Clinical Sciences and Community Health, University of Milan, Milan, Italy; 5 Geriatric Unit, Fondazione IRCCS Ca’ Granda Ospedale Maggiore Policlinico, Milan, Italy; 6 Geriatria, Accettazione Geriatrica e Centro Di Ricerca Per L’invecchiamento, IRCCS INRCA, Ancona, Italy; 7 Laboratory of Clinical and Therapeutical Pharmacology, Lisbon, Portugal; 8 MRC Lifecourse Epidemiology Unit, University of Southampton, Southampton, UK; 9 Servicio de Geriatría. Hospital Universitario Ramón Y Cajal (IRYCIS), Madrid, Spain; 10 Department of Geriatrics, Neurosciences and Orthopedics, Catholic University of the Sacred Heart, Rome, Italy; 11 Scientific Office, Federal Office for Safety in Health Care, Vienna, Austria; 12 CNR Aging Branch-IN, Padua, Italy; 13 State Research Institute Centre for Innovative Medicine, Vilnius, Lithuania; 14 Research Unit of Medical Imaging, Physics and Technology, Faculty of Medicine, University of Oulu, Oulu, Finland; 15 Department of Joint Surgery, The First Affiliated Hospital of Sun Yat-Sen University, Guangzhou, China; 16 Landspitali, University Hospital of Iceland, Reykjavik, Iceland; 17 Department of Diabetes, Nutrition and Metabolic Disorders, Clinical Pharmacology, University of Liege, CHU de Liège, Liège, Belgium; 18 Department of Health Sciences, Vrije Universiteit Amsterdam, Amsterdam, the Netherlands; 19 Agencia Española de Medicamentos Y Productos Sanitarios, Madrid, Spain; 20 Faculty of Medicine, Service of Bone Diseases, Geneva University Hospitals, Geneva, Switzerland

**Correction: Calcifed Tissue International (2023) 112:197–217** **https://doi.org/10.1007/s00223-022-01054-z**

In the article, the affiliation of one of the co-author Antonio Cherubini was incorrect. The correct affiliation is as follows: “Geriatria, Accettazione geriatrica e Centro di ricerca per l’invecchiamento, IRCCS INRCA, Ancona, Italy”.

This has been corrected with this correction.

